# Neurovascular Coupling Remains Intact During Incremental Ascent to High Altitude (4240 m) in Acclimatized Healthy Volunteers

**DOI:** 10.3389/fphys.2018.01691

**Published:** 2018-11-28

**Authors:** Jack K. Leacy, Shaelynn M. Zouboules, Carli R. Mann, Joel D. B. Peltonen, Gurkan Saran, Cassandra E. Nysten, Heidi E. Nysten, Tom D. Brutsaert, Ken D. O’Halloran, Mingma T. Sherpa, Trevor A. Day

**Affiliations:** ^1^Department of Biology, Mount Royal University, Calgary, AB, Canada; ^2^Department of Physiology, School of Medicine, College of Medicine & Health, University College Cork, Cork, Ireland; ^3^Red Deer Regional Hospital, Red Deer, AB, Canada; ^4^School of Education, Syracuse University, Syracuse, NY, United States; ^5^Kunde Hospital, Khumjung, Nepal

**Keywords:** neurovascular coupling, hypoxia, hypocapnia, high-altitude, cerebral blood flow

## Abstract

Neurovascular coupling (NVC) is the temporal link between neuronal metabolic activity and regional cerebral blood flow (CBF), supporting adequate delivery of nutrients. Exposure to high altitude (HA) imposes several stressors, including hypoxia and hypocapnia, which modulate cerebrovascular tone in an antagonistic fashion. Whether these contrasting stressors and subsequent adaptations affect NVC during incremental ascent to HA is unclear. The aim of this study was to assess whether incremental ascent to HA influences the NVC response. Given that CBF is sensitive to changes in arterial blood gasses, in particular PaCO_2_, we hypothesized that the vasoconstrictive effect of hypocapnia during ascent would decrease the NVC response. 10 healthy study participants (21.7 ± 1.3 years, 23.57 ± 2.00 kg/m^2^, mean ± SD) were recruited as part of a research expedition to HA in the Nepal Himalaya. Resting posterior cerebral artery velocity (PCAv), arterial blood gasses (PaO_2_, SaO_2_, PaCO_2_, [HCO_3_^-^], base excess and arterial blood pH) and NVC response of the PCA were measured at four pre-determined locations: Calgary/Kathmandu (1045/1400 m, control), Namche (3440 m), Deboche (3820 m) and Pheriche (4240 m). PCAv was measured using transcranial Doppler ultrasound. Arterial blood draws were taken from the radial artery and analyzed using a portable blood gas/electrolyte analyzer. NVC was determined in response to visual stimulation (VS; Strobe light; 6 Hz; 30 s on/off × 3 trials). The NVC response was averaged across three VS trials at each location. PaO_2_, SaO_2_, and PaCO_2_ were each significantly decreased at 3440, 3820, and 4240 m. No significant differences were found for pH at HA (*P* > 0.05) due to significant reductions in [HCO_3_^-^] (*P* < 0.043). As expected, incremental ascent to HA induced a state of hypoxic hypocapnia, whereas normal arterial pH was maintained due to renal compensation. NVC was quantified as the delta (Δ) PCAv from baseline for mean PCAv, peak PCAv and total area under the curve (ΔPCAv tAUC) during VS. No significant differences were found for Δmean, Δpeak or ΔPCAv tAUC between locations (*P* > 0.05). NVC remains remarkably intact during incremental ascent to HA in healthy acclimatized individuals. Despite the array of superimposed stressors associated with ascent to HA, CBF and NVC regulation may be preserved coincident with arterial pH maintenance during acclimatization.

## Introduction

High altitude (HA) presents several stressors, both environmental and physiological, to normal human function and survival. Environmental stressors can include, but are not limited to, severe winds, cold temperatures and decreases in barometric pressure. Due to reductions in barometric pressure, total oxygen availability decreases. Consequential physiological stressors include hypoxia, ventilatory-induced hypocapnia and acid-base disturbances. These stressors drive several physiological adaptations to preserve homeostasis (e.g., ventilatory acclimatization). This dynamic relationship between stressor and adaptation has inspired scientific research for generations into the exact mechanisms and pathways responsible for maintaining homeostasis and health at HA.

In normal circumstances the human brain accounts for only 2% of total body weight, and yet is responsible for 20% of total energy consumption within the body (Attwell et al., 2011; Bor-Seng-Shu et al., 2011; Subudhi et al., 2014). Brain tissue possesses a high RMR, with limited capacity for glycogen storage (Brown and Ransom, 2007; Willie et al., 2014). Consequently, the cerebrovasculature requires tight, narrow control of CBF for the delivery of nutrients and removal of metabolic wastes (Drake and Iadecola, 2007; Lecrux and Hamel, 2011). The brain maintains adequate CBF during instances of increased neuronal metabolic demand due to high vascularisation and a sophisticated regulation of rCBF (Phillips et al., 2016).

Regarding the cerebrovasculature, many studies have shown that acute exposure to HA induces an increase in gCBF, with values returning to sea-level during acclimatization (Severinghaus et al., 1966; Møller et al., 2002; Lucas et al., 2011). This response stems from the vasodilatory effect of hypoxia below a certain threshold (<40–45 mmHg) (Brugniaux et al., 2007; Ainslie and Ogoh, 2010; Willie et al., 2014; Flück et al., 2015). The increase in gCBF is crucial to maintain CDO_2_ at HA (Hoiland et al., 2016). CDO_2_ is a product of gCBF and arterial oxygen content (Subudhi et al., 2014). gCBF increases during exposure to HA to compensate for the decrease in PaO_2,_ maintaining CDO_2_ (Hoiland et al., 2016).

Although research into the effects of HA on CBF is comprehensive, the impact of ascent to HA on the control of CBF, *per se*, is less well understood. CBF is controlled via three distinct mechanisms: cerebrovascular reactivity to alterations in blood gasses, cerebral autoregulation in response to changes in perfusion pressure, and NVC. NVC pertains to the tight regulation between local neuronal activity and increased regional CBF (rCBF) (Caldwell et al., 2018). NVC is controlled via the interplay between astrocytes, neurons and microvessels within the cerebrovasculature (endothelial cells and pericytes) (Girouard and Iadecola, 2006; Filosa et al., 2016; Venkat et al., 2016). Each of these work in concert with one another by eliciting a vasoactive effect on local microvessels within the cerebrovasculature. A validated method of evoking an NVC response is via VS of the occipital lobe (Phillips et al., 2016). The resultant NVC response can be monitored by assessment of the PCA using TCD (Willie et al., 2011). TCD provides beat-by-beat changes in cerebral blood velocity among other parameters to assess the signal-flow coupling relationship. Acclimatization to HA and its influence on NVC is complex due to multiple contrasting stimuli. Exposure to hypoxia results in hypoxic vasodilation. However, hypoxia triggers the HVR (Teppema and Dahan, 2010). The HVR manifests increased ventilatory drive raising PaO_2_ levels with concomitant decreases in PaCO_2_ (hypocapnia). The resultant hypocapnia is a potent vasoconstrictor (Wilson et al., 2009). The cerebrovasculature is particularly sensitive to changes in ABGs, especially PaCO_2_ (Brugniaux et al., 2007; Ainslie and Ogoh, 2010). Previous literature shows that as little as a 1 mmHg increase or decrease from normal PaCO_2_ levels results in a 3–6% increase or 1–3% decrease in gCBF, respectively (Willie et al., 2014; Phillips et al., 2016). Whether one of these competing stressors elicits a dominant effect on vessel reactivity to stimulus at HA is intriguing as laboratory-based research has shown that hypocapnia reduces NVC response magnitude (Szabo et al., 2011).

The impact of incremental ascent to HA on NVC remains unclear. To our knowledge, only one other study has directly investigated the effect of HA on the NVC response (Caldwell et al., 2017). Caldwell and colleagues observed no significant effects of HA on the NVC response, comparing sea-level measures for NVC to multiple days (3 and 7 days) at one specific altitude (3800 m). We wished to address the issue by way of an incremental ascent profile to a higher altitude (4240 m). We investigated the effects of four progressively increasing altitudes on the NVC response making direct comparisons between altitudes, within-individuals. In addition, we determined ABGs and acid-base status during ascent to characterize the acclimatization process. Given that cerebrovascular tone is superiorly sensitive to changes in PaCO_2_ compared with PaO_2_, we hypothesized that the vasoconstrictive effect of hypocapnia would reduce the NVC response with ascent.

## Materials and Methods

### Participants and Ethical Approval

**FIGURE 1:**
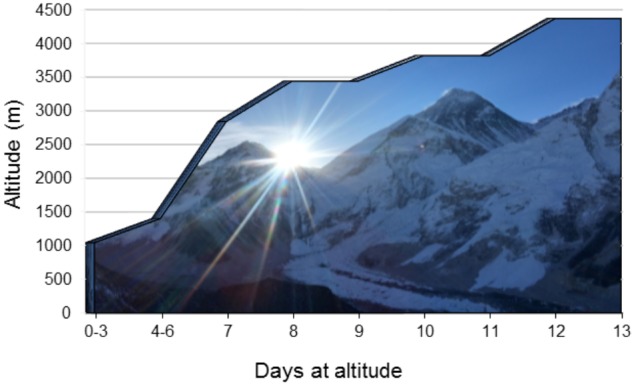
Ascent profile. An illustration of the expedition ascent profile. The days spent at each location are presented on the *x*-axis with the corresponding altitude of each location presented on the *y*-axis. Arrows denote measurement location/dates.

Ten study participants were recruited as part of a 21-day research expedition to Everest base camp in the Nepal Himalaya. The expedition was considered a low-risk ascent profile (Zuckerman, 2012; see **Figure 1** for ascent profile). All participants had no prior-history of cardiovascular, respiratory or cerebrovascular disease. Nine subjects were residents of Calgary (1045 m). One subject was an Irish national (0 m; sea-level) who had been living in Calgary for 5 months prior to the expedition. Several over-the-counter medications were taken during ascent, predominantly as a method of curtailing symptoms associated with exposure to HA (headache, diarrhea, cough, flu-like symptoms). The medications used were: Imodium, Ibuprofen, Neocitran, Advil, Pepto bismal and DM expectant syrup. Several of the female participants were taking oral contraception (Yasmin and Tricylen-lo) during the expedition. Diamox (acetazolamide) was not taken by any participant at any time during the ascent.

This study abided by the Canadian Government Tri-Council policy on research ethics with human participants (TCPS2) and conformed with the standards set by the latest revision of the *Declaration of Helsinki*, except for registration in a database. Ethical approval was received in advance through the Mount Royal University Human Research Ethics Board (Protocols 100012 and 100922) and was harmonized with the Nepal Health Research Council (Protocol 109-2017). All participants were recruited via verbal communication and all provided written and informed consent prior to voluntary participation in the study. Although this study took place in the context of a large research expedition to altitude, the specific study design, research question and data collection were planned *a priori*.

### Physiological Measures

Posterior cerebral artery blood velocity (PCAv) and NVC response were assessed and later compared between Calgary (1045 m), Namche (3440 m), Deboche (3820 m), and Pheriche (4240 m). Similarly, arterial blood gasses (ABGs; PaO_2_, SaO_2_, PaCO_2,_ arterial bicarbonate [HCO_3_^-^], base excess and arterial pH) were collected at the same pre-determined locations as PCAv and NVC. Most baseline ABGs were collected at 1045 m. However, two participants had their baseline ABGs taken at Kathmandu (1400 m) due to logistical issues. Therefore, when describing ABGs within the results section we have referred to 1045 m/1400 m as baseline.

A total of 3 days were spent in Kathmandu (1400 m) prior to beginning the expedition. In addition to NVC, PCAv, and ABGs, several daily measures were obtained each morning, beginning in Kathmandu and continued for the duration of the expedition. The measures collected each morning were: HR, arterial blood pressure (Systolic; SBP, Diastolic; DBP), pressure of end-tidal CO_2_ [(P_ET_)CO_2_], peripheral oxyhemoglobin saturation (SpO_2_), Hb and hematocrit (hct) (see **Table 1**).

**Table 1:** Daily measures.

Daily measures							
	1400 m	3 440 m		3820 m		4240 m	
	Baseline		P-value		P-value		P-value
P(_ATM_)	648	509	–	486	–	454	–
P_I_O_2_ (mmHg)	136	107	–	102	–	95	–
AMS	0 (2)	1 (3)	–	0 (4)	–	1 (3)	–
Weight (kg)	71.31 ± 14.49	70.30 ± 13.69	*P* = 0.160	70.23 ± 13.68	*P* = 0.126	70.23 ± 13.34	*P* = 0.237
BMI (kg/m^2^)	23.57 ± 2.00	23.35 ± 1.84	*P* = 0.757	23.33 ± 1.84	*P* = 0.515	23.34 ± 1.78	*P* = 0.826
Systolic (mmHg)	112.50 ± 10.77	116.40 ± 8.32	*P* = 1.000	119.40 ± 10.98	*P* = 0.154	118.70 ± 10.68^∗^	*P* < 0.001
Diastolic (mmHg)	77.90 ± 11.05	86.60 ± 8.41^∗^	*P* = 0.001	89.60 ± 10.45^∗^	*P* = 0.002	87.40 ± 9.42^∗^	*P* = 0.001
MAP (mmHg)	89.43 ± 10.63	96.39 ± 7.32^∗^	*P* = 0.026	99.53 ± 10.13^∗^	*P* = 0.006	97.83 ± 9.27^∗^	*P* < 0.001
HR (bpm)	85.90 ± 8.85	91.60 ± 13.92	*P* = 0.473	84.10 ± 7.49	*P* = 0.100	84.90 ± 10.90	*P* = 0.100
SpO_2_ (%)	96.40 ± 0.97	91.80 ± 2.49^∗^	*P* = 0.005	91.40 ± 2.84^∗^	*P* = 0.005	87.60 ± 2.01^∗†^	*P* = 0.005
P(_ET_)CO_2_ (Torr)	32.60 ± 6.77	26.50 ± 2.99^∗^	*P* = 0.043	25.30 ± 2.67^∗^	*P* = 0.018	21.10 ± 1.91^∗†¥^	*P* = 0.001

*Daily measures collected each day, beginning in Kathmandu (1400 m). BMI, body mass index; MAP, mean arterial pressure; HR, heart rate; SpO_*2*_, peripheral oxygen saturation; P(_*ET*_)CO_*2*_, partial pressure of end-tidal CO_*2*_; P(_*ATM*_), total atmospheric pressure; P_*I*_O_*2*_, partial pressure of inspired oxygen; AMS, acute mountain sickness score. The exact p-value comparing each altitude to baseline is provided. ^∗^ = Statistical significance from 1400 m. ^†^ = Statistical significance from 3440 m. ¥ = Statistical significance from 3820 m. NS = non-significant (P > 0.05). Presented as mean ± SD, AMS data are presented as median (range).*

**Table 2:** Arterial blood gas and acid-base status.

Arterial blood draws							
	1045/1400 m	3440 m		3820 m		4240 m	
	Baseline		*P*-value		*P*-value		*P*-value
PaO_2_ (mmHg)	84.80 ± 5.35	48.56 ± 8.08^∗^	*P* < 0.001	54.56 ± 6.23^∗^	*P* < 0.001	48.38 ± 4.96^∗^	*P* < 0.001
SaO_2_ (%)	96.80 ± 0.79	84.89 ± 7.87^∗^	*P* = 0.032	88.78 ± 3.73^∗^	*P* = 0.003	85.50 ± 3.66^∗^	*P* < 0.001
PaCO_2_ (mmHg)	34.84 ± 4.58	30.23 ± 4.93^∗^	*P* = 0.015	29.57 ± 3.76^∗^	*P* = 0.016	29.05 ± 3.73^∗^	*P* = 0.001
Bicarbonate (mmol/L)	23.24 ± 2.69	20.99 ± 3.08	*P* = 0.417	19.48 ± 2.01^∗^	*P* = 0.043	19.69 ± 2.80^∗†^	*P* = 0.021
Base excess (mmol/L)	–1.20 ± 2.78	–3.00 ± 3.08	*P* = 1.000	–4.78 ± 1.86	*P* = 0.077	–4.50 ± 3.07^†^	*P* = 1.000
pH	7.43 ± 0.02	7.45 ± 0.02	*P* = 0.879	7.43 ± 0.02^†^	*P* = 1.000	7.44 ± 0.02	*P* = 1.000

*Changes in arterial blood values with ascent. The exact p-value comparing each altitude to baseline is provided. ^∗^ = denotes statistical significance from 1045/1400 m. ^†^ = Statistical significance from 3440 m. Presented as mean ± SD.*

During ascent, participants spent a total of 2 days at each rest day altitude (3440, 3820, and 4240 m). The 1st day consisted of arrival having trekked from the preceding lower altitude, with recovery from the trek and one night’s sleep at the new altitude. The 2nd day consisted of data collection and an overnight stay before further ascent beginning the following morning. PCAv, NVC and ABGs were measured on the 2nd day at each altitude. This was implemented to reduce the possible effects of recent exercise on each variable. Participants were also tested sequentially in an identical fashion for PCAv and NVC at each location to account for the possible effects of circadian rhythm on cerebral hemodynamics.

### Instrumentation

#### Daily Measures and Arterial Blood Draws

Each morning (06:00–08:00), non-invasive fasted daily measures were made including weight (digital scale; Omron, model OMRHBF514C), Hb concentration (hemoglobinometer; Hemocue HB201+) and hematocrit (hct; heparinized capillary tube, mini-centrifuge; StatSpin, CritSpin microhematocrit system, Model M960). The participant was seated in a private and quiet room and provided with white noise through head phones to minimize distraction. Subsequently, respiratory rate and (P_ET_)CO_2_ were measured using a portable, calibrated capnograph (Masimo EMMA, Danderyd, Sweden) with a personal mouthpiece and nose clip. HR and SpO_2_ were measured with a portable finger pulse oximeter (Masimo SET^®^ Rad-5, Danderyd, Sweden). Arterial blood pressure (i.e., systolic, diastolic) was measured using an automated blood pressure cuff (Omron, model BP786n) and used to calculate pulse pressure (systolic-diastolic) and MAP (1/3 systolic + 2/3 diastolic). Arterial blood draws were taken and assessed using a blood gas analyzer (Abbott iStat, CG4+ and CHEM 8+ cartridges; Mississauga, Ontario, Canada; blood gasses corrected for altitude and body temperature; see **Table 2**). Each participant filled out the Lake Louise AMS questionnaire to assess self-reported AMS symptoms (see **Table 1**).

#### TCD Instrumentation

A non-invasive measurement of instantaneous HR was obtained using an ECG (lead II configuration; ADInstruments Bioamp ML132; Colorado Springs, CO, United States). PCAv was measured using a Spencer Technologies 2 Mhz TCD (model MD150B; Redmond, WA, United States) using standardized procedures for vessel identification (see Willie et al., 2011). NVC was tested using VS (iPhone “Strobe Light” app). Data was recorded, and later analyzed offline using the PowerLab software (v8.0) provided by ADInstruments (model 16SP ML880) and LabChart Pro software 8.0 (Colorado Springs CO, United States).

### Experimental Protocol

#### NVC Protocol

**FIGURE 2:**
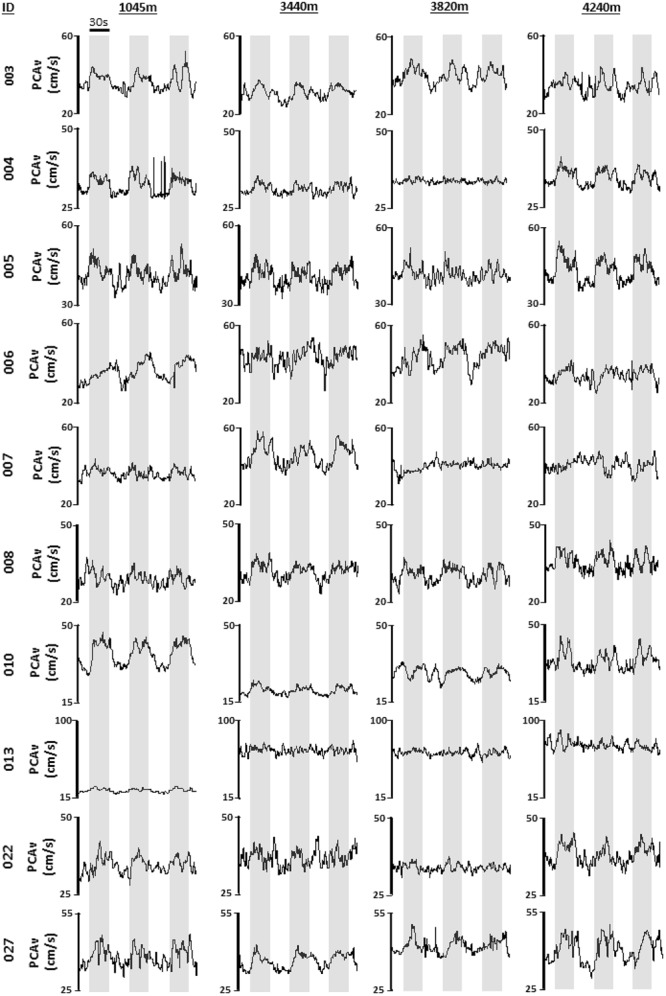
Individual mean PCAv. Original tracings of derived mean PCAv for each participant, during each visual stimulation (VS) trial, at each altitude are provided. Mean PCAv is presented along the *y*-axis with time presented along the *x*-axis. Gray shaded regions denote thirty second periods of VS.

Immediately after daily measures had been completed participants reported to the designated room for NVC testing. Participants were tested in a quiet and dark room in a seated position. The duration of the study protocol was approximately 30 min. Participants were first instrumented with the ECG in lead II configuration. A pulse oximeter was placed on the participant’s finger to measure beat-by-beat peripheral oxyhemoglobin saturation for the duration of the protocol. The TCD headpiece was fixed on the participant’s head to a comfort level determined by the participant. The headpiece was utilized to avoid probe movement during the protocol. Ultrasound probes were fixed at either side of the participant’s head on the *trans*-temporal window. The *trans*-temporal window is located approximately 1 cm in front of the external auditory meatus and roughly 1–2 cm above the zygomatic arch. The P2 segment of the PCA was insonated and monitored throughout. Standard methods for determining the PCA vessel were applied prior to beginning the study (e.g., vessel depth and resting velocity, flow direction, visual responsiveness and carotid compression; Willie et al., 2011). Following set-up, the participant sat in a resting state with the room lights switched off and eyes closed for a period of 3 min. This period formed the basis of baseline PCAv measures. Following the 3-min baseline period, NVC was assessed via a standardized VS (strobe light; 6 Hz). The strobe light (iPhone application “Strobe light”) was placed approximately six inches in front of the participant’s eyes. Participants were exposed to three consecutive trials of VS (30 s on eyes open/30 s off eyes closed). Participants were monitored continuously to ensure that their eyes remained open during VS. Once VS had been completed this signaled the end of the NVC assessment. This protocol was repeated at each altitude, using the same vessel. Original recordings for each individual NVC response during VS for each participant at all four altitudes is shown in **Figure 2**.

#### Arterial Blood Draws

Arterial blood samples were obtained from the radial artery by a trained and registered Respiratory Therapist (HN) using standard procedures and universal precautions. Blood samples were collected with the participant in the supine position.

### Data and Statistical Analysis

#### Daily Measures, Arterial Blood Draws, PCAv and NVC

Daily measures (HR, SBP, DBP, MAP, P_ET_CO_2_, and SpO_2_) were recorded as absolute values and compared between locations. Similarly, values for PaO_2_, SaO_2_, PaCO_2_, arterial [HCO_3_^-^], base excess and arterial blood pH were taken as absolute values and compared between locations. AMS scores (Roach et al., 1993) were recorded at each location and are presented as median (range) (see **Table 1**). Approximate atmospheric pressure (P_ATM_) at each altitude was obtained online^1^ (Baillie, 2010) with partial pressure of inspired O_2_ (P_I_O_2_) calculated by multiplying P_ATM_ by 0.21. Baseline PCAv was taken as an average across a 3-min period immediately prior to NVC testing. Baseline total area under the curve for PCAv (PCAv tAUC) was calculated across a 30-s period immediately prior to the onset of NVC assessment. To assess for any baseline drift during the protocol, comparisons were made both for PCAv and PCAv tAUC between baseline and the rest periods between trials. No significant differences were found for either baseline PCAv or PCAv tAUC between the pre-trial baseline and the rest periods between trials (*P* > 0.05). Three separate parameters were used to quantify the NVC response. First, the mean PCAv achieved during the entirety of each 30 s VS was calculated, averaged across the three trials and then compared with baseline PCAv. This was ascribed ΔMean PCAv. Second, the peak PCAv achieved during each VS was identified, averaged across the three trials and then compared with baseline PCAv; this was ascribed ΔPeak PCAv. Finally, PCAv tAUC achieved during each VS was calculated, averaged across the three trials and compared with baseline PCAv tAUC. This was ascribed ΔPCAv tAUC. The NVC response is presented both as absolute change from baseline and percentage increase from baseline. This methodological approach was replicated and compared between altitudes. Furthermore, to ensure that the NVC response was evoked at each altitude, we present comparisons for each of the defined parameters of NVC at baseline and during each individual VS.

#### Statistical Analysis

All data were analyzed and compared using SPSS (IBM statistics, version 24.0). Data were first tested for normal distribution and sphericity using the Shapiro–Wilk test and Mauchly’s test of sphericity, respectively. In situations of normal distribution, between- and within-location comparisons were made using a one-factor repeated measure analysis of variance (ANOVA) with a Bonferroni *post hoc* adjustment. Statistical significance was assumed at *P* < 0.05. When the assumption of normal distribution was violated, a non-parametric Friedman test of differences among repeated measures was conducted with a Wilcoxon rank test *post hoc* analysis between and within locations. Statistical significance was set at *P* < 0.05 divided by the number of comparisons made.

## Results

### Demographics and Daily Measures

Analysis revealed no significant effect of altitude on weight, BMI and HR (*P* > 0.05, see **Table 1**). Further analysis revealed a significant effect of altitude on SBP (*F*_1.891,17.017_ = 3.232, *P* = 0.067 *N*_P_^2^ = 0.264), where SBP was significantly higher at 4240 m, compared with 1400 m (119 ± 11 vs. 113 ± 11 mmHg, mean ± SD, respectively; *P* < 0.001; see **Table 1**). A significant effect of altitude was observed for DBP (*F*_3,27_ = 19.39, *P* < 0.001 *N*_P_^2^ = 0.683), with DBP being significantly elevated at 3440, 3820, and 4240 m compared with 1400 m (87 ± 8, 90 ± 10 and 87 ± 9 vs. 78 ± 11 mmHg, respectively; *P* < 0.001, *P* = 0.002, and *P* < 0.001, respectively; see **Table 1**). Similarly, a significant effect of altitude was observed in MAP (*F*_3,27_ = 13.613, *P* < 0.001 *N*_P_^2^ = 0.602). MAP was elevated at 3440, 3820, and 4240 m compared with 1400 m (96 ± 7, 100 ± 10, and 98 ± 9 vs. 89 ± 11 mmHg, respectively; *P* = 0.026, *P* = 0.006, and *P* < 0.001, respectively; see **Table 1**). A significant effect of altitude was observed in (P_ET_)CO_2_ (*F*_1.246,11.214_ = 25.012, *P* < 0.001 *N*_P_^2^ = 0.735). (P_ET_)CO_2_ was significantly lower at 3440, 3820, and 4240 m compared with 1400 m (26.5 ± 3.0, 25.3 ± 2.7, and 21.1 ± 1.9 vs. 32.6 ± 6.8 Torr, respectively; *P* = 0.043, *P* = 0.018, and *P* < 0.001, respectively; see **Table 1**). Furthermore, (P_ET_)CO_2_ was significantly lower at 4240 m compared with both 3440 and 3820 m (*P* < 0.001 and *P* < 0.001, respectively). Finally, a significant effect of altitude was observed for SpO_2_ [*X*^2^(3) = 25.906, *P* < 0.001]. SpO_2_ was significantly lower at 3440, 3820, and 4240 m compared with 1400 m (91.80 ± 2.49, 91.40 ± 2.84, and 87.60 ± 2.01 vs. 96.40 ± 0.97%, respectively; *P* = 0.005, *P* = 0.005, and *P* = 0.005, respectively; see **Table 1**). Furthermore, SpO_2_ was significantly lower at 4240 m compared with 3440 m (*P* = 0.008).

### Arterial Blood Gas and Acid-Base Status

**FIGURE 3:**
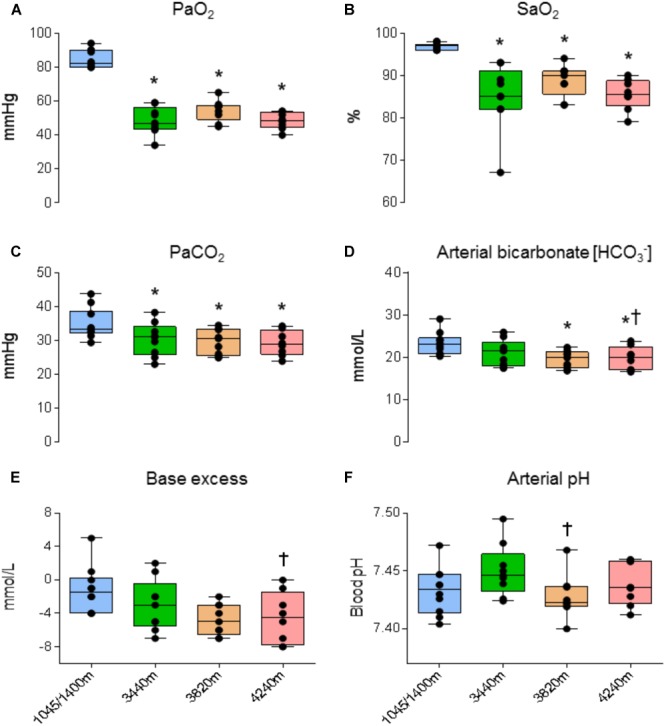
Arterial blood gas and acid-base data. Ascent to high altitude (HA) had a significant effect on **(A)** PaO_2_ (*P* < 0.001), **(B)** SaO_2_ (*P* < 0.032), **(C)** PaCO_2_ (*P* < 0.016), **(D)** arterial bicarbonate [HCO_3_^-^] (*P* < 0.043). Compared with baseline, ascent to HA had no significant effect on **(E)** base excess (*P* > 0.05) and **(F)** arterial pH (*P* > 0.05). ^∗^ = denotes statistical significance from 1045/1400 m. † = Statistical significance from 3440 m. Presented as scatter plots representing individual participants with box and whiskers showing median, 25–75 percentile with minimum and maximum values.

Analysis revealed a statistically significant effect of altitude on PaO_2_ (*F*_3,18_ = 138.270, *P* < 0.001 *N*_p_^2^ = 0.958). PaO_2_ was significantly lower at 3440, 3820, and 4240 m compared with 1045/1400 m (48.5 ± 8.1, 54.6 ± 6.2, 48.4 ± 5.0 vs. 84.8 ± 5.4 mmHg, respectively; *P* < 0.001, *P* < 0.001, and *P* < 0.001, respectively; see **Figure 3A**). A statistically significant effect of altitude on SaO_2_ (*F*_3,18_ = 17.313, *P* < 0.001 *N*_p_^2^ = .743). SaO_2_ was significantly lower at 3440, 3820, and 4240 m compared with 1045/1400 m (84.9 ± 7.9, 88.8 ± 3.7, 85.5 ± 3.7 vs. 96.8 ± 0.8%, respectively; *P* = 0.032, *P* = 0.003, and *P* < 0.001, respectively; see **Figure 3B**). A statistically significant effect of altitude on PaCO_2_ (*F*_3,18_ = 23.123, *P* < 0.001 *N*_p_^2^ = 0.794). PaCO_2_ was significantly lower at 3440, 3820, and 4240 m compared with 1045/1400 m (30.2 ± 4.9, 29.6 ± 3.8, 29.1 ± 3.7 vs. 34.8 ± 4.6 mmHg, respectively; *P* = 0.015, *P* = 0.016, and *P* < 0.001, respectively; see **Figure 3C**). A statistically significant effect of altitude on [HCO_3_^-^] (*F*_3,18_= 11.342, *P* < 0.001, *N*_p_^2^ = 0.654). [HCO_3_^-^] was significantly lower at 3820 and 4240 m compared with 1045/1400 m (19.5 ± 2.0, 19.7 ± 2.8 vs. 23.2 ± 2.7 mmol/L, respectively; *P* = 0.043 and *P* = 0.021, respectively; see **Figure 3D**). No significant differences were found between 1045/1400 m and 3440 m for [HCO_3_^-^] (23.2 ± 2.7 vs. 21.0 ± 3.1 mmol/L, respectively; *P* = 0.417), but a significant difference for [HCO_3_^-^] was found between 3440 and 4240 m (*P* = 0.006). A statistically significant effect of altitude on base excess (*F*_3,18_ = 7.132, *P* = 0.002, *N*_p_^2^ = 0.543). Base excess was significantly lower at 4240 m compared with 3440 m (-4.143 ± 1.18 vs. -2.286 ± 1.19 mmol/L, respectively; *P* = 0.022; see **Figure 3E**). Analysis observed no statistically significant effect of altitude on arterial pH when compared with baseline (*F*_3,18_ = 2.788, *P* = 0.070 *N*_p_^2^ = 0.317; see **Figure 3F**).

### Baseline PCAv and PCAv tAUC Comparisons

**FIGURE 4:**
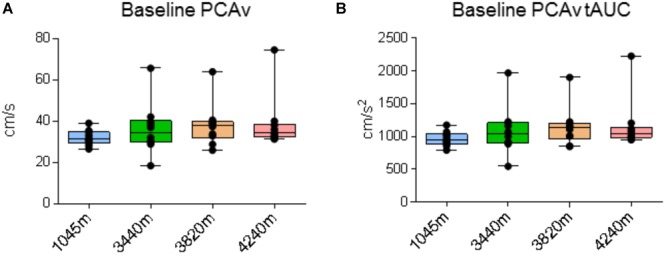
Cerebral blood velocity. Data for posterior cerebral artery velocity (PCAv) during baseline recording. Ascent to HA had no significant effect on **(A)** baseline PCAv (*P* > 0.131) and **(B)** baseline PCAv total area under the curve (tAUC) (*P* > 0.086). Presented as scatter plots representing individual participants with box and whiskers showing median, 25–75 percentile with minimum and maximum values.

There were no significant differences for baseline PCAv or baseline PCAv tAUC between locations [*X*^2^(3) = 5.640, *P* = 0.131 and *X*^2^(3) = 6.60, *P* = 0.086, respectively; see **Figures 4A,B**, respectively].

### NVC Response (ΔMean PCAv, ΔPeak PCAv or ΔPCAv tAUC)

We first sought to assert that the NVC response was evoked at each location. We therefore compared the absolute mean and peak PCAv achieved during each individual VS to baseline PCAv. We replicated this analysis for PCAv tAUC, thus covering all three NVC parameters.

#### 1045 m

**FIGURE 5:**
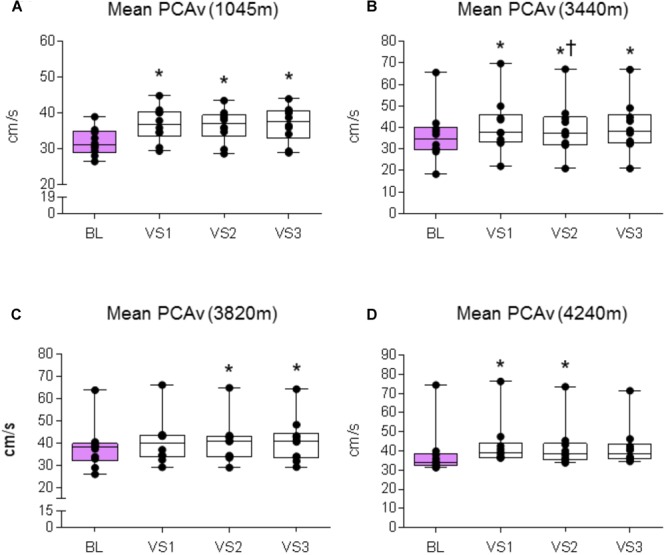
Mean PCAv response. Comparison of the mean PCAv achieved during baseline (BL) and during each individual VS at each altitude. Significant differences were found between BL PCAv and mean PCAv during VS at **(A)** 1045 m (*P* < 0.003), **(B)** 3440 m (*P* < 0.010), **(C)** 3820 m (*P* < 0.005), and **(D)** 4240 m (*P* < 0.007). ^∗^ = Statistical significance from BL. † = Statistical significance from VS 1. Presented as scatter plots representing individual participants with box and whiskers showing median, 25–75 percentile with minimum and maximum values.

**FIGURE 6:**
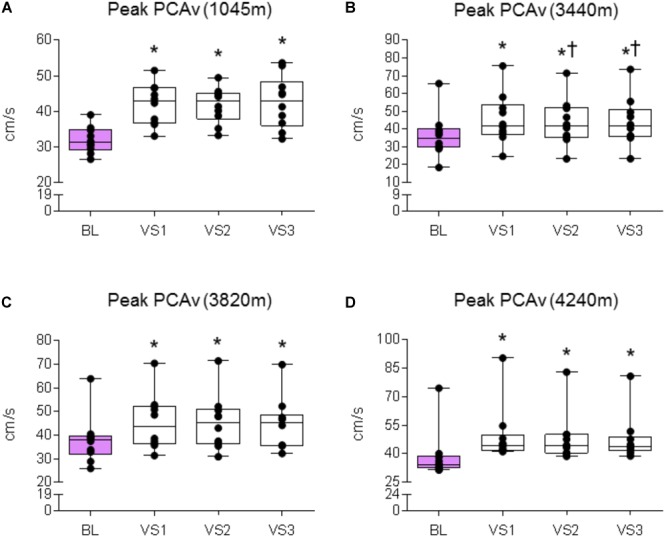
Peak PCAv response. Comparison of the mean PCAv during BL and peak PCAv during each individual VS. Significant differences were found between BL PCAv and peak PCAv during VS at **(A)** 1045 (*P* < 0.001), **(B)** 3440 m (*P* < 0.001), **(C)** 3820 m (*P* < 0.007), and **(D)** 4240 m (*P* < 0.005). ^∗^ = Statistical significance from BL. † = Statistical significance from VS 1. Presented as scatter plots representing individual participants with box and whiskers showing median, 25–75 percentile with minimum and maximum values.

**FIGURE 7:**
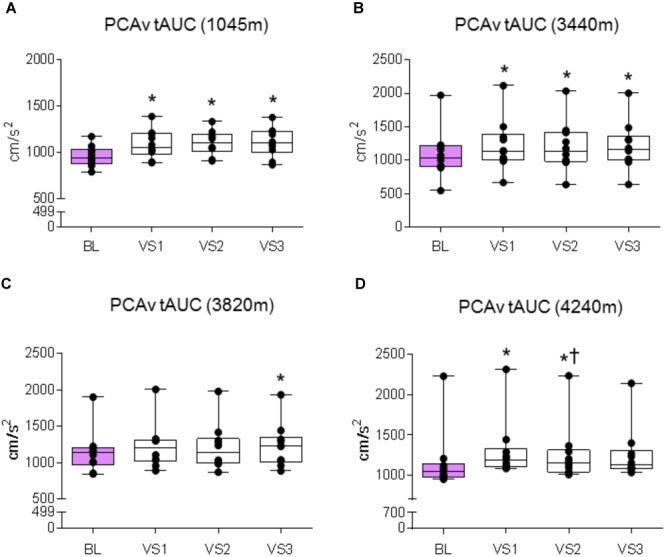
PCAv tAUC response. Comparison of the posterior cerebral artery velocity total area under the curve (PCAv tAUC) achieved during BL and PCAv tAUC during each individual VS. Significant differences were found between BL PCAv tAUC and PCAv tAUC during VS at **(A)** 1045 m (*P* < 0.006), **(B)** 3440 m (*P* < 0.003), **(C)** 3820 m (*P* = 0.005), and **(D)** 4240 m (*P* < 0.005). ^∗^ = Statistical significance from BL. † = Statistical significance from VS 1. Presented as scatter plots representing individual participants with box and whiskers showing median, 25–75 percentile with minimum and maximum values.

A significant effect was found between baseline and VS for mean PCAv, peak PCAv, and PCAv tAUC (*F*_1.5,13.503_ = 29.151, *P* < 0.001, *N*_p_^2^ = 0.764; *F*_3,27_ = 42.513, *P* < 0.001, *N*_p_^2^ = 0.825; *F*_3,27_ = 15.847, *P* < 0.001, *N*_p_^2^ = 0.638, respectively). Further analysis revealed that mean PCAv, peak PCAv, and PCAv tAUC were statistically different during each individual VS compared with baseline (*P* < 0.003, *P* < 0.001, and *P* < 0.006, respectively; see **Figures 5A, 6A, 7A**).

#### 3440 m

A significant effect was found between baseline and VS for mean PCAv, peak PCAv and PCAv tAUC (*F*_1.385,12.466_ = 20.913, *P* < 0.001, *N*_p_^2^ = 0.699; *F*_1.414,12.723_ = 48.820, *P* < 0.001, *N*_p_^2^ = 0.844; *F*_3,27_ = 22.749, *P* < 0.001, *N*_p_^2^ = 0.717, respectively). Further analysis revealed that mean PCAv, peak PCAv, and PCAv tAUC were statistically different during each individual VS compared with baseline (*P* < 0.010, *P* < 0.001, and *P* = 0.003, respectively; see **Figures 5B, 6B, 7B**). Additionally, *post hoc* analysis found a statistically significant difference between mean PCAv during VS 1 and VS 2 (*P* = 0.034). Furthermore, a statistically significant difference was observed between peak PCAv during VS 1 compared with VS 2 and VS 3 (*P* = 0.027 and *P* = 0.016, respectively).

#### 3820 m

There was a statistically significant effect observed for mean PCAv, peak PCAv, and PCAv tAUC between baseline and VS [*X*^2^(3) = 16.80, *P* < 0.001; *X*^2^(3) = 17.64, *P* < 0.001; and *X*^2^(3) = 15.00, *P* = 0.002, respectively]. Further analysis revealed that mean PCAv was significantly higher during VS 2 and VS 3 compared with baseline (BL) (*P* = 0.005, *P* = 0.005, respectively; see **Figure 5C**) and that peak PCAv was significantly higher during each VS trial compared with BL (*P* < 0.007; see **Figure 6C**). PCAv tAUC was significantly higher during VS 3 only, compared with BL (*P* = 0.005; see **Figure 7C**).

#### 4240 m

There was a statistically significant effect observed for mean PCAv, peak PCAv, and PCAv tAUC between baseline and VS [*X*^2^(3) = 16.44, *P* < 0.001; *X*^2^(3) = 19.56, *P* < 0.001; and *X*^2^(3) = 20.52, *P* < 0.001, respectively]. *Post hoc* analysis revealed that mean PCAv was significantly higher during VS 1 and VS 2 compared with baseline (BL) (*P* = 0.005, *P* = 0.007, respectively; see **Figure 5D**). Additionally, peak PCAv was significantly higher during each VS trial compared with BL (*P* < 0.005; see **Figure 6D**). PCAv tAUC was significantly higher during VS 1 and VS 2 compared with BL (*P* = 0.005, *P* = 0.005, respectively; see **Figure 7D**). Furthermore, *post hoc* analysis revealed a significant effect between VS 1 and VS 2 for PCAv tAUC (*P* = 0.007).

#### Between Location NVC Comparison

**FIGURE 8:**
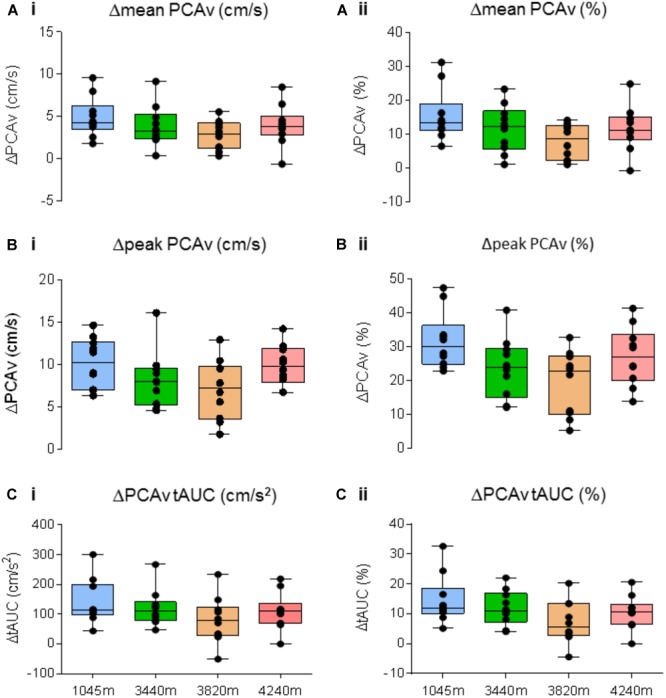
Neurovascular coupling (NVC) response. Comparison of the NVC response for Δmean PCAv, Δpeak PCAv, and ΔPCAv tAUC between altitudes. The magnitude of the response is presented as both the respective Δ from baseline (left column), and the corresponding percentage change from baseline (right column). No significant differences were found for **(A)**Δmean PCAv (*P* > 0.05), **(B)**Δpeak PCAv (*P* > 0.05), **(C)**ΔPCAv tAUC (*P* > 0.05). Presented as scatter plots representing individual participants with box and whiskers showing median, 25–75 percentile with minimum and maximum values.

There was no significant effect of altitude on either Δmean PCAv, Δpeak PCAv, or ΔPCAv tAUC (*F*_3,27_ = 1.948, *P* = 0.146, *N*_p_^2^ = 0.178, *F*_3,27_ = 1.948, *P* = 0.045, *N*_p_^2^ = 0.254 and *F*_3,27_ = 1.384, *P* = 0.269, *N*_p_^2^ = 0.133, respectively; see **Figures 8A–C**, respectively).

## Discussion

### Primary Findings

This study investigated the influence of incremental ascent to 4240 m over 7 days on NVC. Our results demonstrate: (1) the NVC response was evoked consistently at each altitude, and (2) there was no significant difference in the magnitude of the NVC response at the level of the PCA between altitudes in healthy acclimatized individuals.

### Acclimatization

Exposure to HA and the associated drop in total barometric pressure induced a state of hypoxemia. This is evident from the significant decreases in PaO_2_ and SaO_2_ (see **Figures 3A,B**). During prolonged exposure to HA, hypoxia increases activation of the peripheral chemoreceptors, enhancing the HVR (Ainslie and Ogoh, 2010; Willie et al., 2015; Hoiland et al., 2016). The HVR increases ventilatory drive partially restoring PaO_2_ and SaO_2_ levels toward resting values (Bernardi et al., 2006; Smith et al., 2014). Comparing values recorded at 3440m with those obtained at higher altitudes, with further decreases in barometric pressure, we observed no further reductions in PaO_2_ and SaO_2_, revealing attenuation of a further decline in oxygenation associated with continued ascent.

Secondly, we observed profound hypocapnia, as demonstrated by significant decreases in PaCO_2_, indicative of hyperventilation (see **Figure 3C**). Hypocapnia serves to blunt both central and peripheral chemoreceptor activation and subsequently reduces ventilatory drive. Combined, these data reveal the presence of two antagonistic vasoactive stressors: hypoxia and hypocapnia. Hypoxia, below a threshold (<40–45 mmHg), has a vasodilatory effect in the systemic circuit (Ainslie and Ogoh, 2010). In contrast, hypocapnia is a potent vasoconstrictor (Brugniaux et al., 2007). The cerebrovasculature is particularly sensitive to changes in ABGs, especially PaCO_2_ (Brugniaux et al., 2007; Ainslie and Ogoh, 2010). Although prior research has shown that the presence of hypocapnia decreases the NVC response (Szabo et al., 2011), this study was laboratory-based and did not include the hypoxic vasodilatory stress associated with HA. Furthermore, the presence of sustained hypocapnia presents further issues by inducing tissue alkalosis (Krapf et al., 1991).

Hyperventilation-induced hypocapnia triggers an acid-base adjustment in the form of renal compensation. To counteract respiratory alkalosis, and maintain pH within normal limits, a series of compensatory steps are required through increased acid retention coupled with an increase in HCO_3_^-^ excretion via the renal system (Zouboules et al., 2018). The end-product of a relative metabolic acidosis helps to decrease arterial pH toward normal values, thereby preserving acid-base homeostasis (Goldfarb-Rumyantzev and Alper, 2014). The sophisticated and refined nature of this compensatory adaptation is crucial as the vasoconstrictive effects of hypocapnia/alkalosis can decrease CBF (Szabo et al., 2011). Furthermore, a blunted HVR response has been shown to be a strong predictor of AMS (Richalet et al., 2012). We observed evidence of renal compensation by way of significant decreases in arterial [HCO_3_^-^] (see **Figure 3D**) in response to hypocapnia, such that arterial pH was maintained constant throughout ascent compared with BL.

### Impact of HA on CBF and NVC

We found no significant differences in baseline PCAv and baseline PCAv tAUC during ascent (**Figure 4**). In terms of NVC, we observed that for each of our designated NVC parameters, a significant response was evoked during VS (see **Figures 5–7**). Our results demonstrate the remarkable reproducibility of the within-group NVC response. For between-altitude comparisons, our findings demonstrate that NVC remained intact at the level of the PCA, as demonstrated by no significant differences in each of the three NVC response parameters between altitudes (**Figure 8**), although we observed both inter-subject variability of the NVC response and intra-subject variability in the NVC response between altitudes (**Figure 2**). These findings are consistent with a previous study (Caldwell et al., 2017), where NVC testing was performed temporally in response to acclimatization at a single altitude. These studies differed in terms of ascent-profile, altitude reached, analysis and stimulus to evoke NVC. However, the finding that NVC remains intact during ascent to HA is consistent.

### Methodological Limitations

The utilization of TCD for the assessment of CBF has limitations. Although TCD provides excellent beat-by-beat measurement of the intracranial arteries arising from the circle of Willis, it does not provide any indication of pre-existing diameter of the insonated vessel. This is relevant at HA where there are multiple and competing stressors, which influence vessel tone (hypoxic vasodilation and hypocapnic vasoconstriction). There is also recent literature to suggest that there are stimulus-evoked changes in diameter of the PCA as a function of distance from the primary visual cortex (Bizeau et al., 2017). There is therefore an inherent risk of misinterpreting the magnitude of the CBF change (Caldwell et al., 2017). Furthermore, there is a risk of technical error given the sensitivity of transcranial Doppler technique. To account for this, we ensured the same sonographers were present during each testing session. We also strictly abided by a set of guidelines for insonating the PCA (see Willie et al., 2011). We also acknowledge that TCD provides measurements of velocity rather than flow. However, recent literature has demonstrated CBF velocity to be an appropriate and reliable surrogate for CBF (Secher et al., 2008; Ainslie and Ogoh, 2010). Although our findings demonstrate an intact NVC response during incremental ascent to HA, further research is required to fully explore this issue. The cerebrovasculature can be subdivided into three categories: large pial arteries which run along the surface of the cortex (i.e., PCA), pial arterioles which penetrate the cortex and finally parenchymal arterioles and capillaries (Filosa et al., 2016; Phillips et al., 2016). It has been postulated that NVC-mediated responses are initiated within the parenchymal arterioles and capillaries (Filosa et al., 2016). Whether the NVC response within the microvasculature is affected during incremental ascent to HA remains unknown. Although we did not directly measure the NVC response at the microvascular level, the application of TCD to conduit arteries for assessment of NVC in human volunteers is a well-established technique (Willie et al., 2011; Phillips et al., 2016). If the diameter of the PCA was increased during VS, this would have evoked a blunted hyperemic response, that is the increase in PCA diameter would have caused a reduction in PCAv (Phillips et al., 2016). As this was not observed in our study, we assume a constant PCA diameter during VS, with the increase in PCAv most likely due to downstream vasodilation at the level of the neurovascular unit.

## Conclusion

As expected, exposure to HA elicited an incremental state of hypoxic hypocapnia. However, respiratory alkalosis was adequately combatted via renal compensation (relative metabolic acidosis), preserving arterial pH within normal limits during the ascent profile. Despite the combination of stressors associated with incremental ascent to 4240 m, NVC remains remarkably intact through the PCA. We postulate that during exposure to HA, NVC could be predominantly sensitive to arterial pH. This study highlights the remarkable innate ability of the cerebrovasculature to adapt to environmental stress through a series of integrative adaptations, with several intrinsically complex systems working in unison to preserve cerebrovascular homeostasis and function. Strengths of this study include the comprehensive manner by which the NVC response was presented, analyzed and compared at each altitude. Although we tested NVC at a higher altitude than that employed in previous studies (Caldwell et al., 2017), future studies might incorporate even higher altitudes where HA illness symptomology becomes increasingly prevalent. Whereas our study demonstrates that NVC remains intact during incremental ascent to 4240 m in acclimatized volunteers, it is plausible to suggest that responses may be impaired in unacclimatized individuals, during rapid ascent, or perhaps at altitudes higher than 4240 m. It would be interesting to investigate the temporal effects of exposure to HA on NVC, both in the acute and chronic setting.

It will also be important to investigate in other experimental models the effects of chronic hypobaric hypoxia on the NVC response within sub-cortical parenchymal arterioles and capillaries, which is not feasible in field studies at high altitude in humans.

## Author Contributions

Data collection for PCAv and NVC was completed by JL, CM, JP, GS, and TD. Data collection for daily measures was completed by SZ, CN, KO’H, and TB. Arterial blood draws were completed by HN. Safe ascent to and descent from 4240 m was carried out by MS. Arterial blood draw analysis was completed by SZ. PCAv and NVC analysis/interpretation was completed by JL, KO’H, and TD. Manuscript, figure, and table development/preparation was completed by JL, KO’H and TD.

## Conflict of Interest Statement

The authors declare that the research was conducted in the absence of any commercial or financial relationships that could be construed as a potential conflict of interest.

## Footnotes

1 http://altitude.org/

## Abbreviations

BGs: arterial blood gases
AMS: acute mountain sickness
BMI: body mass index
CBF: cerebral blood flow
CDO_2_: cerebral oxygen delivery
DBP: diastolic blood pressure
ECG: electrocardiogram
gCBF: global cerebral blood flow
HA: high altitude
Hb: hemoglobin
HCO_3_^-^: bicarbonate
Hct: hematocrit
HR: heart rate
HVR: hypoxic ventilatory response
MAP: mean arterial pressure
NVC: neurovascular coupling
P(_ET_)CO_2_: partial pressure of end-tidal carbon dioxide
PaCO_2_: partial pressure of arterial carbon dioxide
PaO_2_: partial pressure of arterial oxygen
P_ATM_: total atmospheric pressure
PCA: posterior cerebral artery
PCAv: posterior cerebral artery velocity
P_I_O_2_: Partial pressure of inspired oxygen
rCBF: regional cerebral blood flow
RMR: resting metabolic rate
SaO_2_: arterial oxygen saturation
SBP: systolic blood pressure
SpO_2_: Peripheral oxygen saturation
tAUC: total area under the curve
TCD: transcranial doppler ultrasound
VS: visual stimulation
